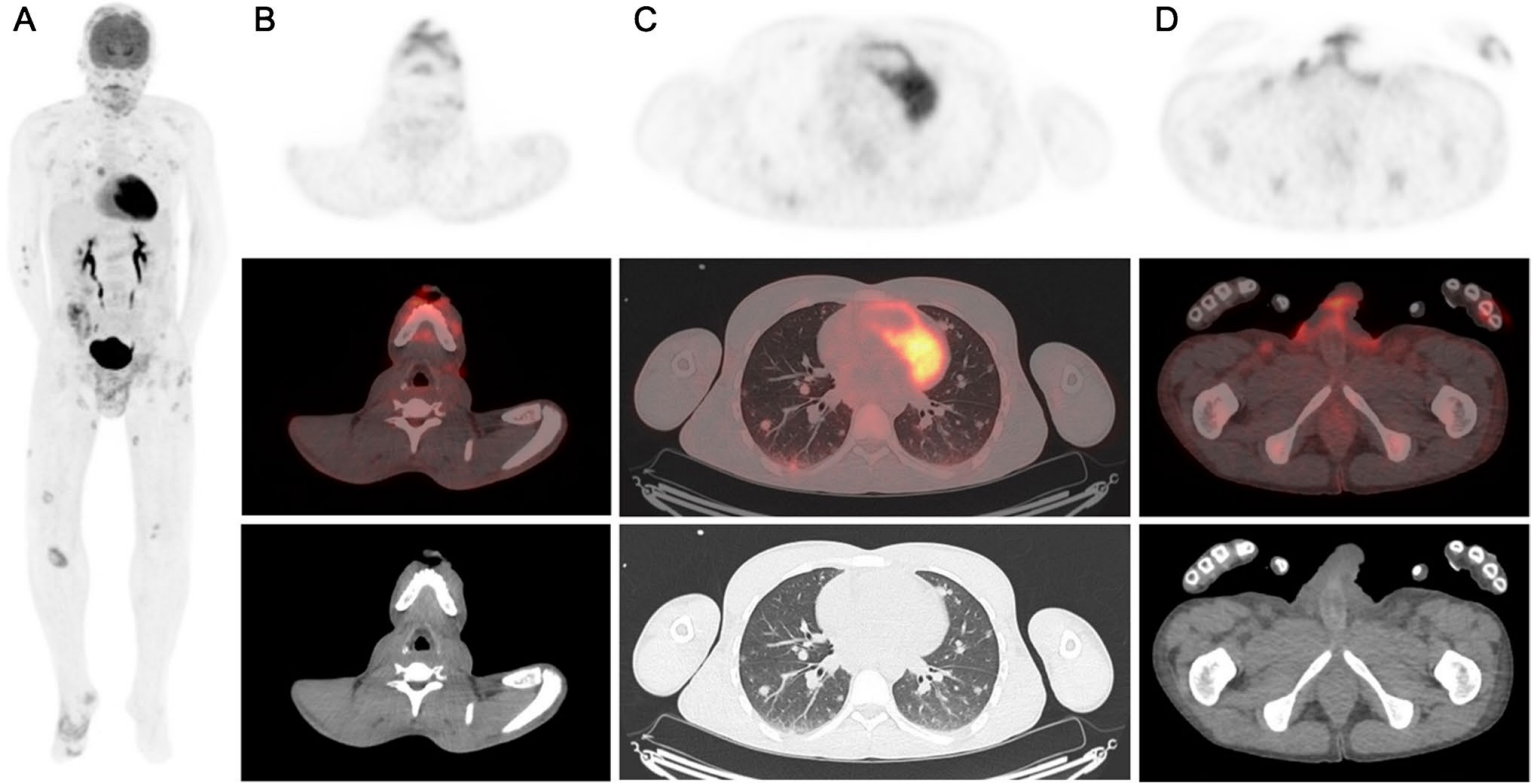# First [18F]-FDG-PET/CT images of a patient infected with Monkeypox

**DOI:** 10.1007/s00259-022-06023-0

**Published:** 2022-11-05

**Authors:** Ringo Manta, Raoul Muteganya, Nicolas Gohimont, Benjamin Heymans, Diana Ene

**Affiliations:** 1 Department of Nuclear Medicine, CHU Saint Pierre, Université Libre de Bruxelles (ULB), Brussels, Belgium; 2 Department of Infectious Diseases, CHU Saint Pierre, Université Libre de Bruxelles (ULB), Brussels, Belgium

A 30-year-old man presented painful disseminated skin and genital lesions with pyrexia. The lesions appeared 1 month prior to the admission as vesicles, progressively evolving into confluent and crusted lesions. Polymerase chain reaction (PCR) assay of the lesions swab was positive for monkeypox and negative for herpes simplex virus. Laboratory investigations showed elevated C-reactive protein and lymphopenia. Human immunodeficiency virus 1/2 antibody/antigen test was reactive. CD4 count was 23.9 cells/mm^3^. Chest radiograph revealed multiple lung opacities. Serology was negative for syphilis, cryptococcus, toxoplasmosis, and positive for cytomegalovirus (CMV) but CMV PCR was negative. Sputum and bronchoalveolar lavage (BAL) were negative for tuberculosis and pneumocystis. [18F]-FDG PET/CT was performed for the detection of a clinically occult AIDS-related disease. Maximal intensity projection images (A) and fused axial images (B, C, D) revealed multiple hypermetabolic cutaneous lesions on the scalp, face, and limbs with corresponding skin thickening on CT; a large hypermetabolic pubic lesion extending in the subcutaneous tissues and scrotum; and numerous lung nodules with mild [18F]-FDG uptake. Mildly hypermetabolic peripheral lymph nodes were also observed. Lung nodules were compatible with viral lung disease occurring in immunocompromised patients [1, 2]. Serology, sputum, and BAL results did not suggest another etiology for this nodular pattern. Lung nodules were hence assumed as Monkeypox-related. Patterns of inflammation in lungs and of immune activation in lymph nodes on PET/CT have been previously described in non-human primates [3, 4]. To our knowledge, this is the first case of disseminated monkeypox disease with skin and lung lesions shown on [18F]-FDG PET/CT.